# Supplementary material for: Leveraging body dielectric polarization for ambient electromagnetic energy recovery via e-textile
Source: Nat Commun. 2025 Oct 9;16:9001. doi: 10.1038/s41467-025-64053-2 (PMC12511592; doi:10.1038/s41467-025-64053-2)
Supplement: Supplementary file 1 — Supplementary Information [file 41467_2025_64053_MOESM1_ESM.pdf]

Supplementary Information for

Leveraging body dielectric polarization for ambient electromagnetic energy recovery via e-textile

Yuanlong Li†, Weifeng Yang †*, Alexander V. Shokurov, Carlo Menon*

Biomedical and Mobile Health Technology Laboratory, Department of Health Sciences and Technology, ETH Zurich, Lengghalde 5, 8008 Zurich, Switzerland.

†These authors contributed equally to this work.

*Corresponding author: Weifeng Yang (weifeng.yang@hest.ethz.ch), Carlo Menon (carlo.menon@hest.ethz.ch)

Supplementary Figures

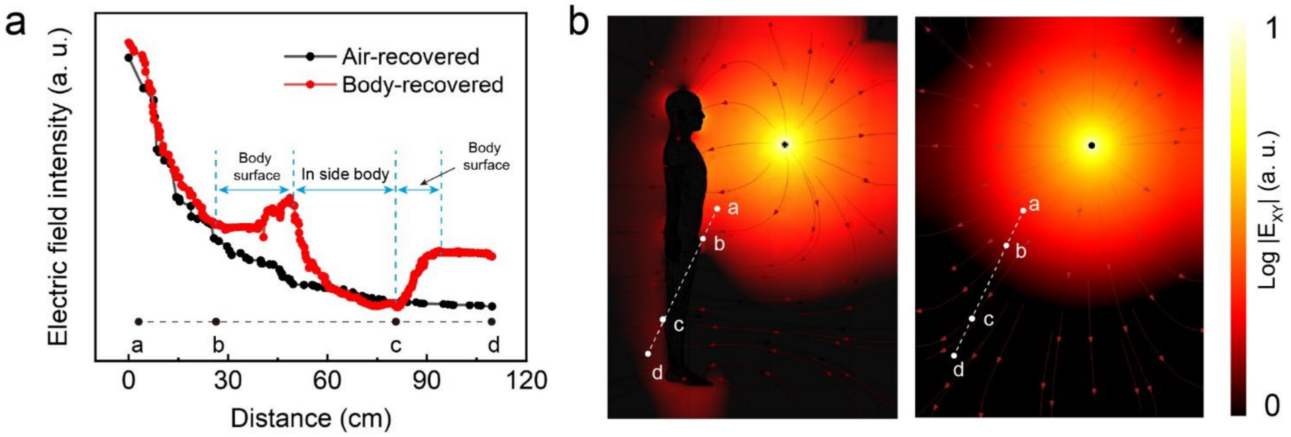

Supplementary Figure 1. COMSOL electric field simulation of EM energy recovered by human body and air. (a) The curve of the recycled electric field passing through the human body. **(b)** Straight line through the body for electric field simulation.

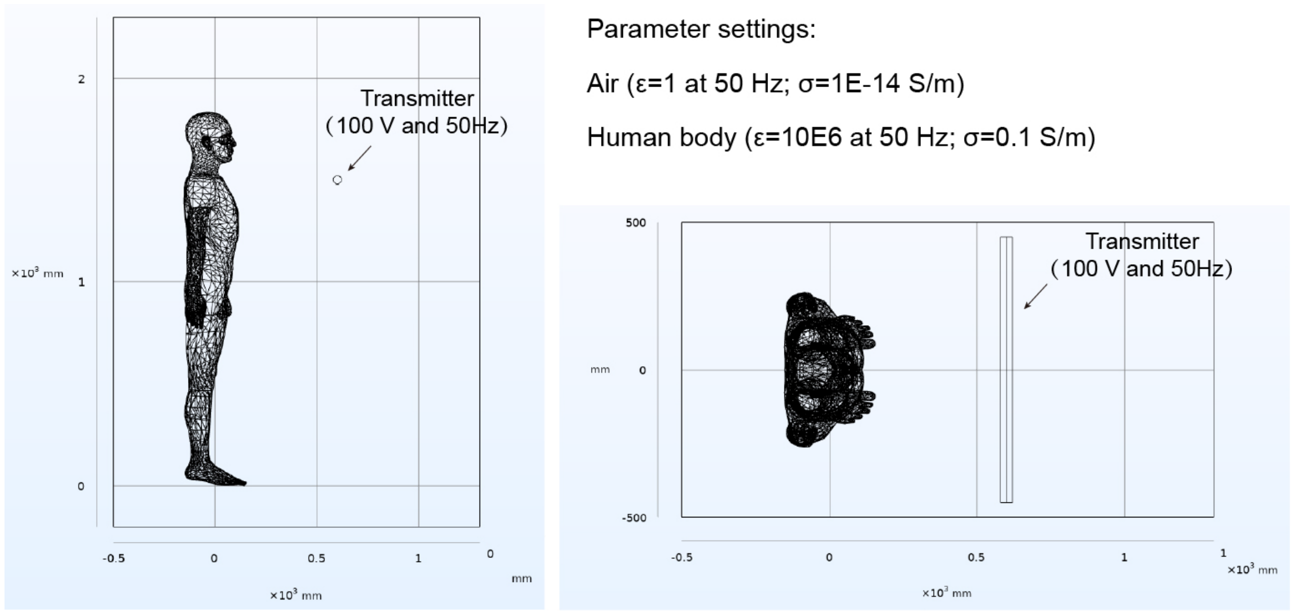

Supplementary Figure 2. COMSOL simulation parameter settings of EM energy recovered by the human body.

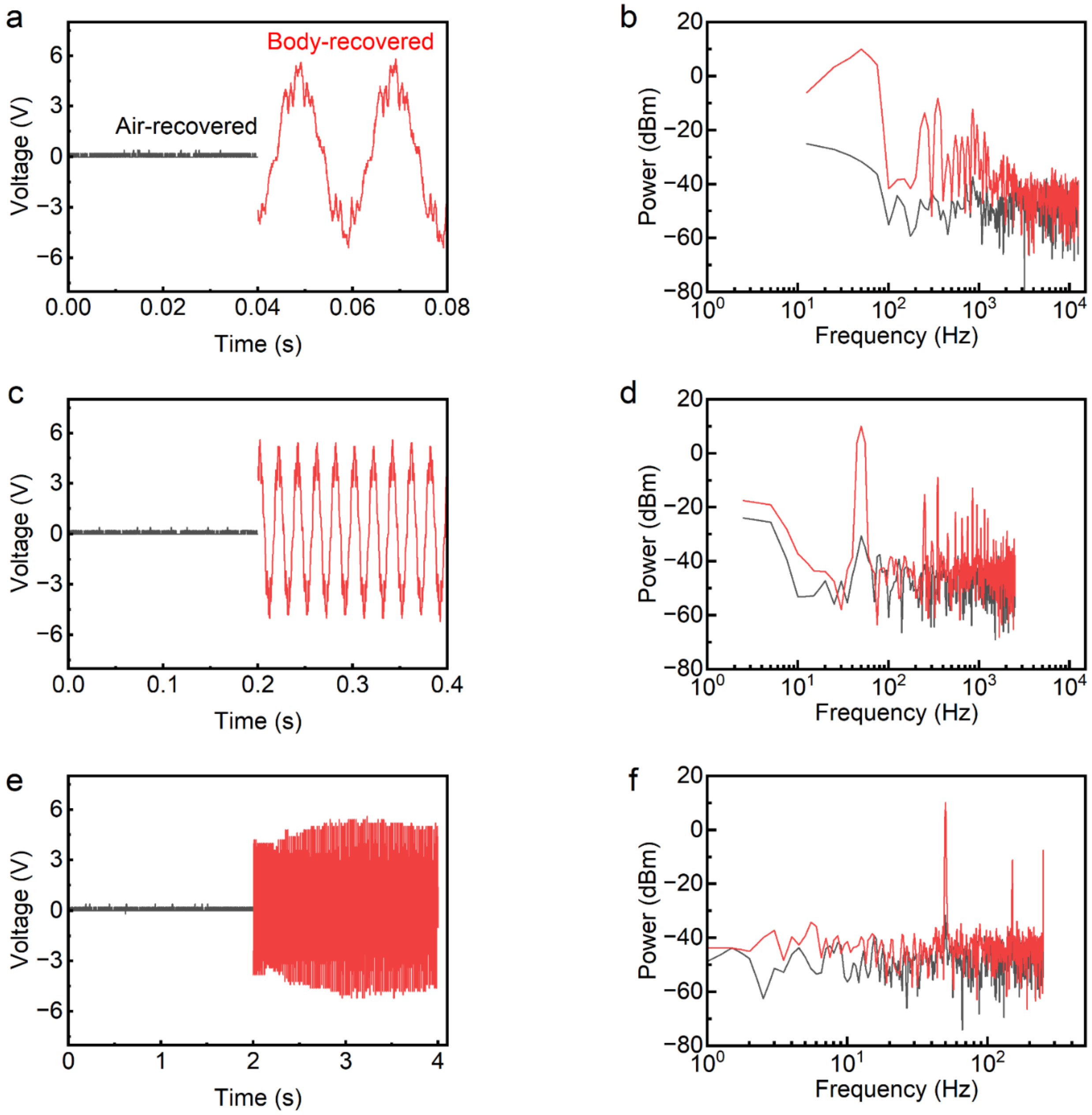

Supplementary Figure 3. Comparison of EM energy recovery at different temporal resolutions. **(a)** voltage and **(b)** frequency-domain signals based on air/body recovery in 0.04 seconds' period. **(c)** voltage and **(d)** frequency-domain signals based on air/body recovery in 0.2 seconds' period. **(e)** voltage and **(f)** frequency-domain signals based on air/body recovery in 2 seconds' period.

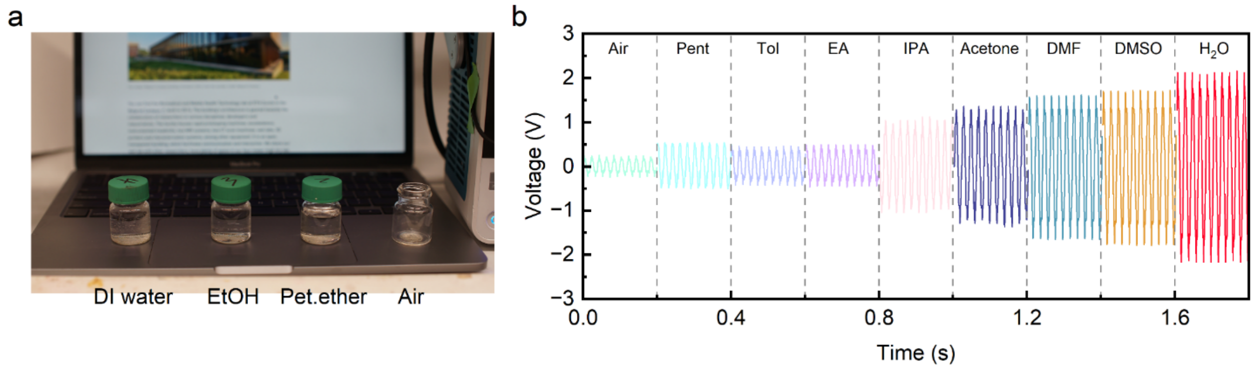

Supplementary Figure 4. Comparison of EM energy recovery in solvents with different polarities. (a) Schematic diagram of the testing scenario for different solvents. **(b)** Comparison of the output voltage recovered by different reagents in this environment.

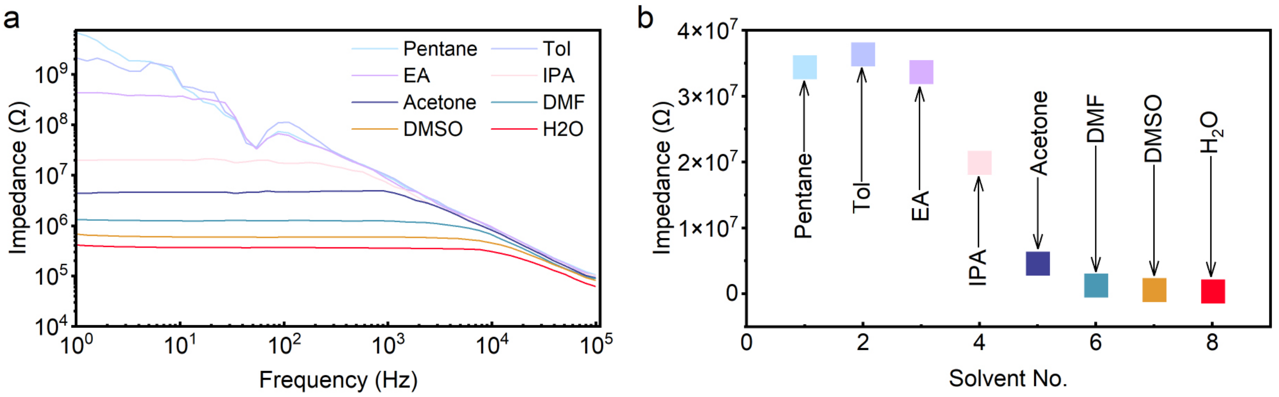

Supplementary Figure 5. Impedance measurement of solvents with different polarities. (a) Impedance of different solvents in the frequency range of 1~10^5 Hz. **(b)** Impedance of different solvents at 50 Hz.

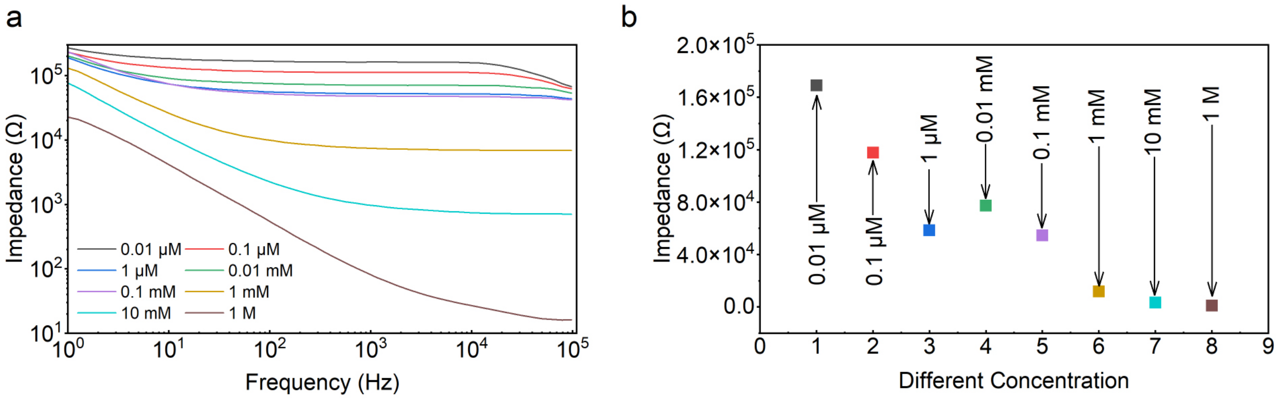

Supplementary Figure 6. Impedance measurement of sodium chloride solutions with different concentrations. (a) Impedance of different solvents in the frequency range of 1~10^5 Hz. **(b)** Impedance of different solvents at 50 Hz.

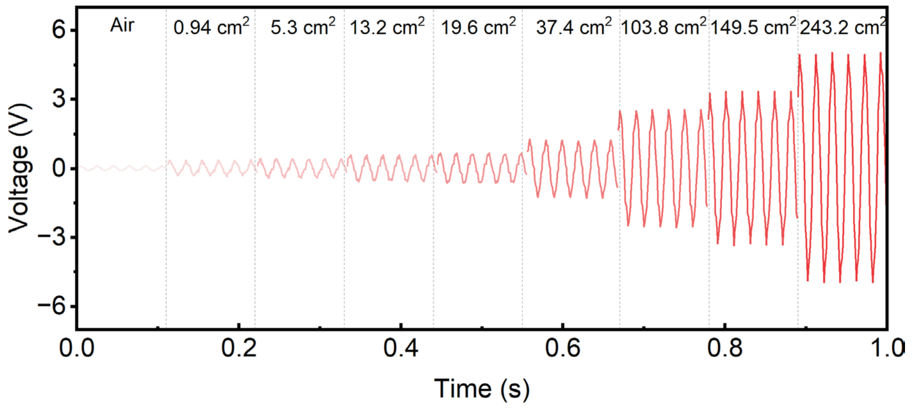

Supplementary Figure 7. Comparison of the output voltage recovered under different contact areas in an electromagnetic field environment. Similar results were obtained for PolaTex samples in three independent tests.

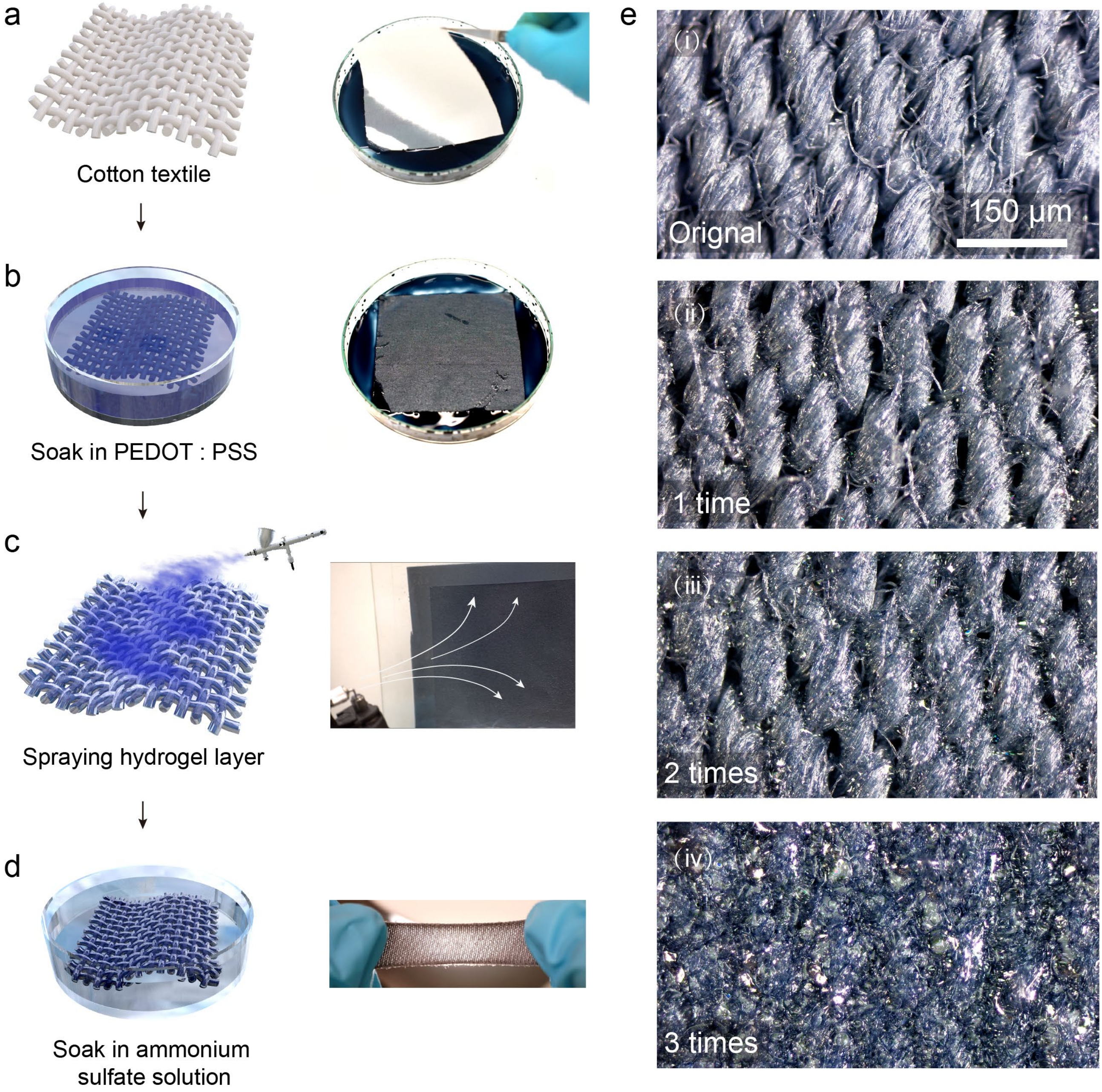

Supplementary Figure 8. Schematic diagram of the fabrication process of PolaTex. (a) Pure cotton fabric **(b)** Cotton fabric was immersed in a PEDOT: PSS solution, forming an electrically conductive layer on the surface. **(c)** After PEDOT: PSS treatment, a layer of hydrogel was applied to the cotton surface using air-spray deposition. **(d)** The sprayed PolaTex was immersed in 20 wt% ammonium sulfate solution. **(e)** Microscopic surface structure of cotton at different deposition spray times. The images provided here are representative of micrographs obtained from three samples.

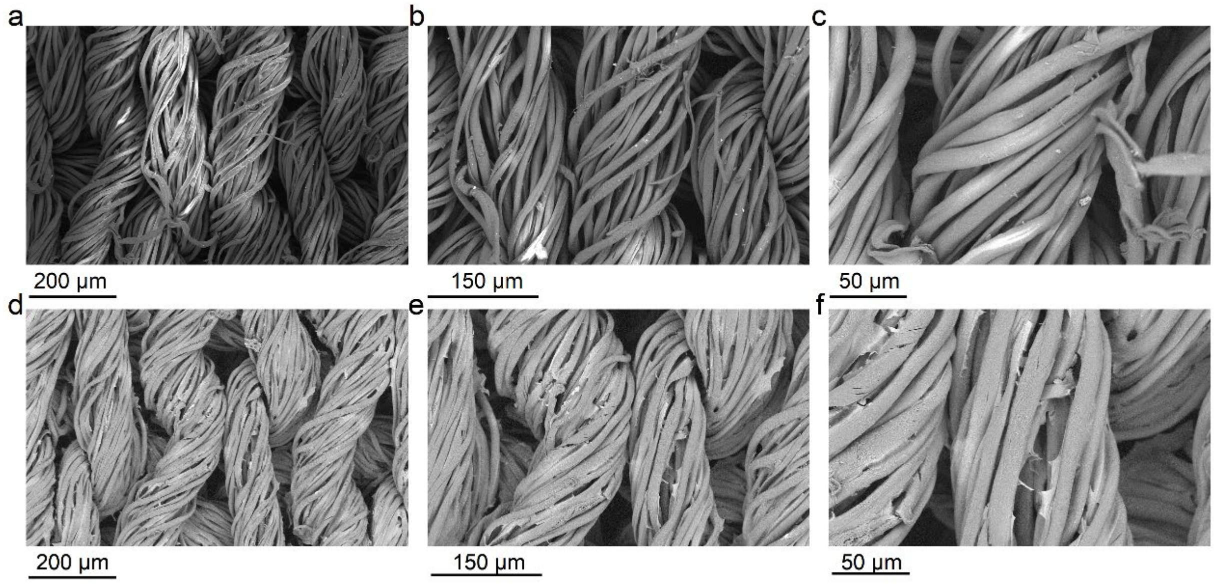

Supplementary Figure 9. The condition of the cotton surface (a-c) before and (d-f) after PEDOT: PSS treatment. The images provided here are representative of micrographs obtained from three samples.

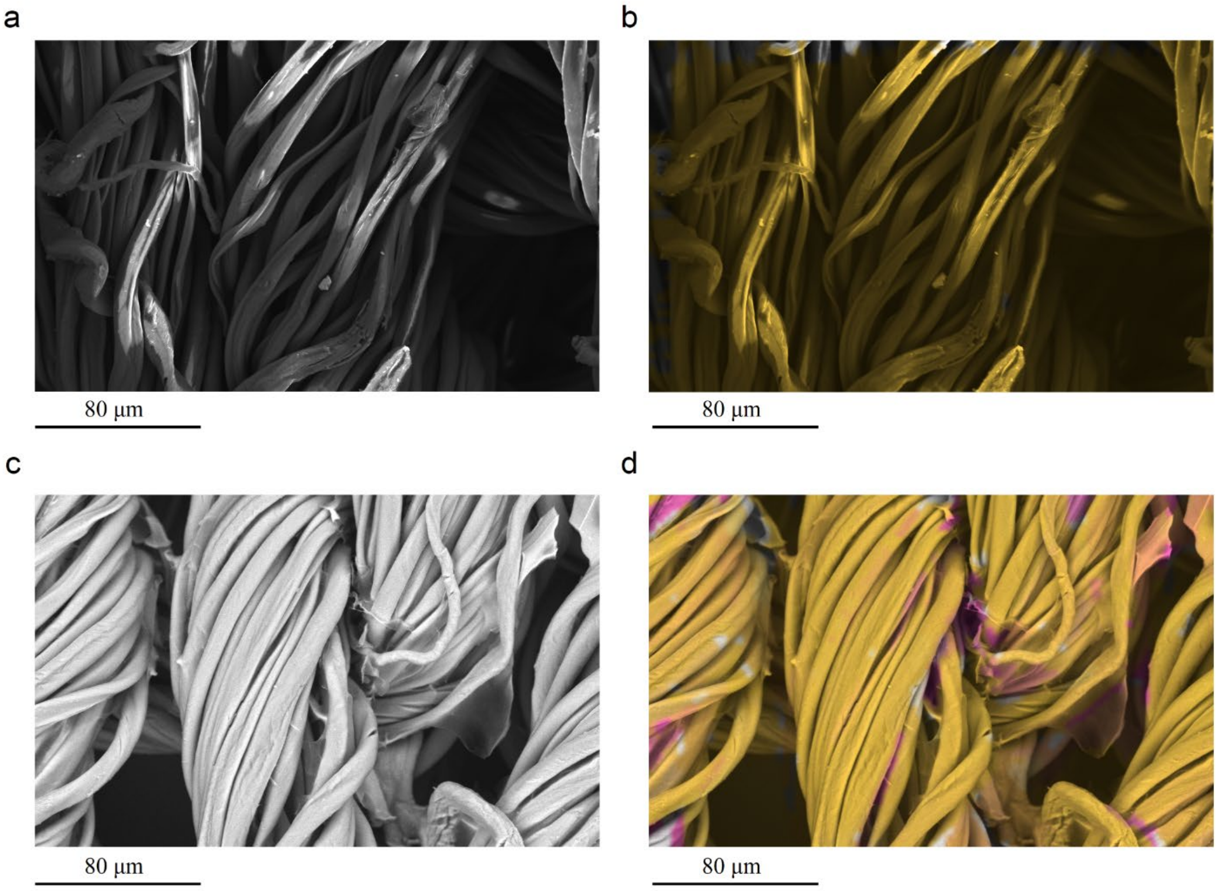

Supplementary Figure 10. The mapping of the cotton surface (a) before and (b) after PEDOT: PSS treatment. The images provided here are representative of micrographs obtained from three samples.

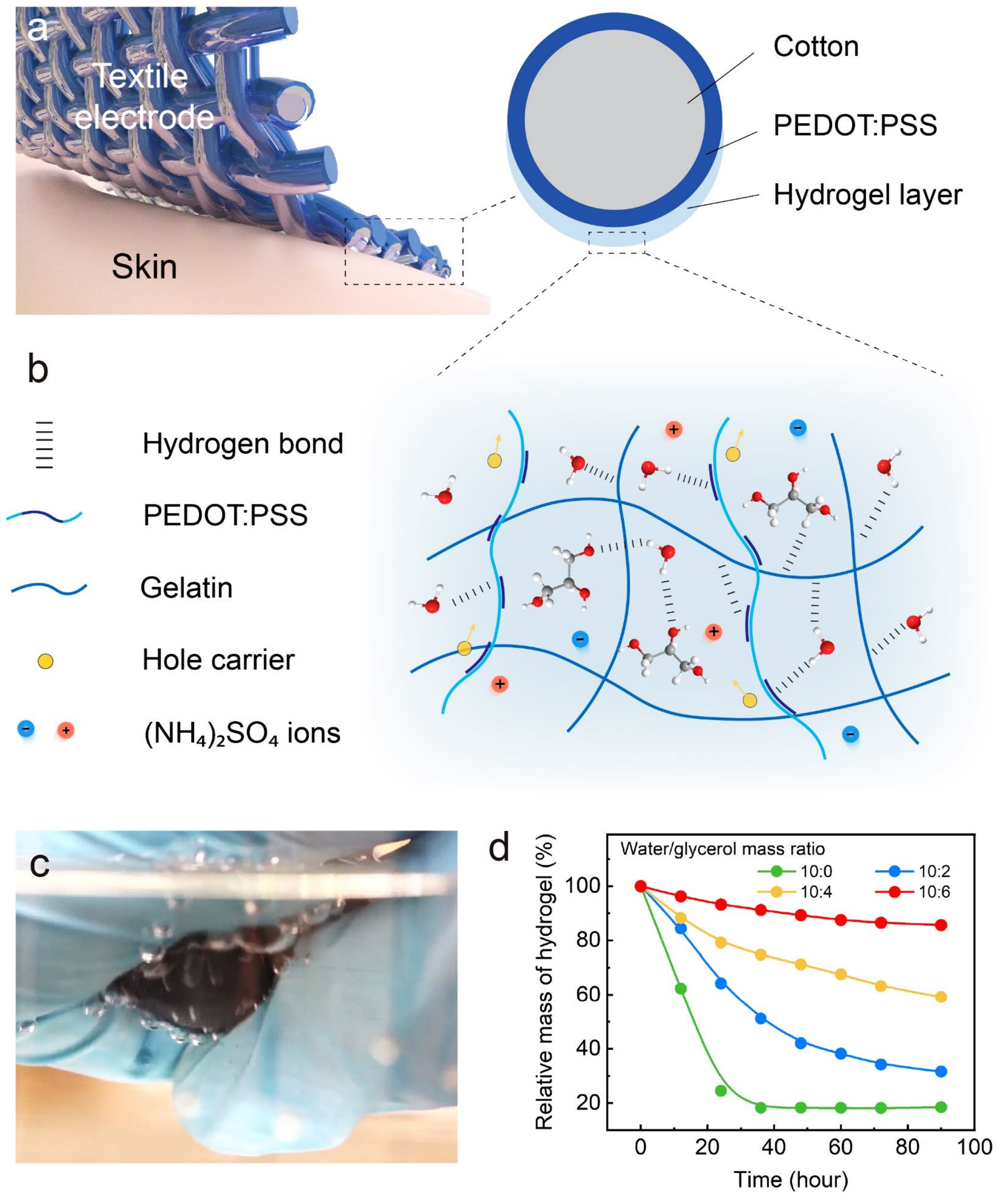

Supplementary Figure 11. Structure and breathability performance of the PolaTex. (a) Schematic diagram of the structure of PolaTex. **(b)** Ionic structure diagram of gelatin-based hydrogel. **(c)** Breathability test of PolaTex. **(d)** Water retention test of PolaTex with different water/glycerol ratios. Similar results were obtained for PolaTex samples in three independent tests.

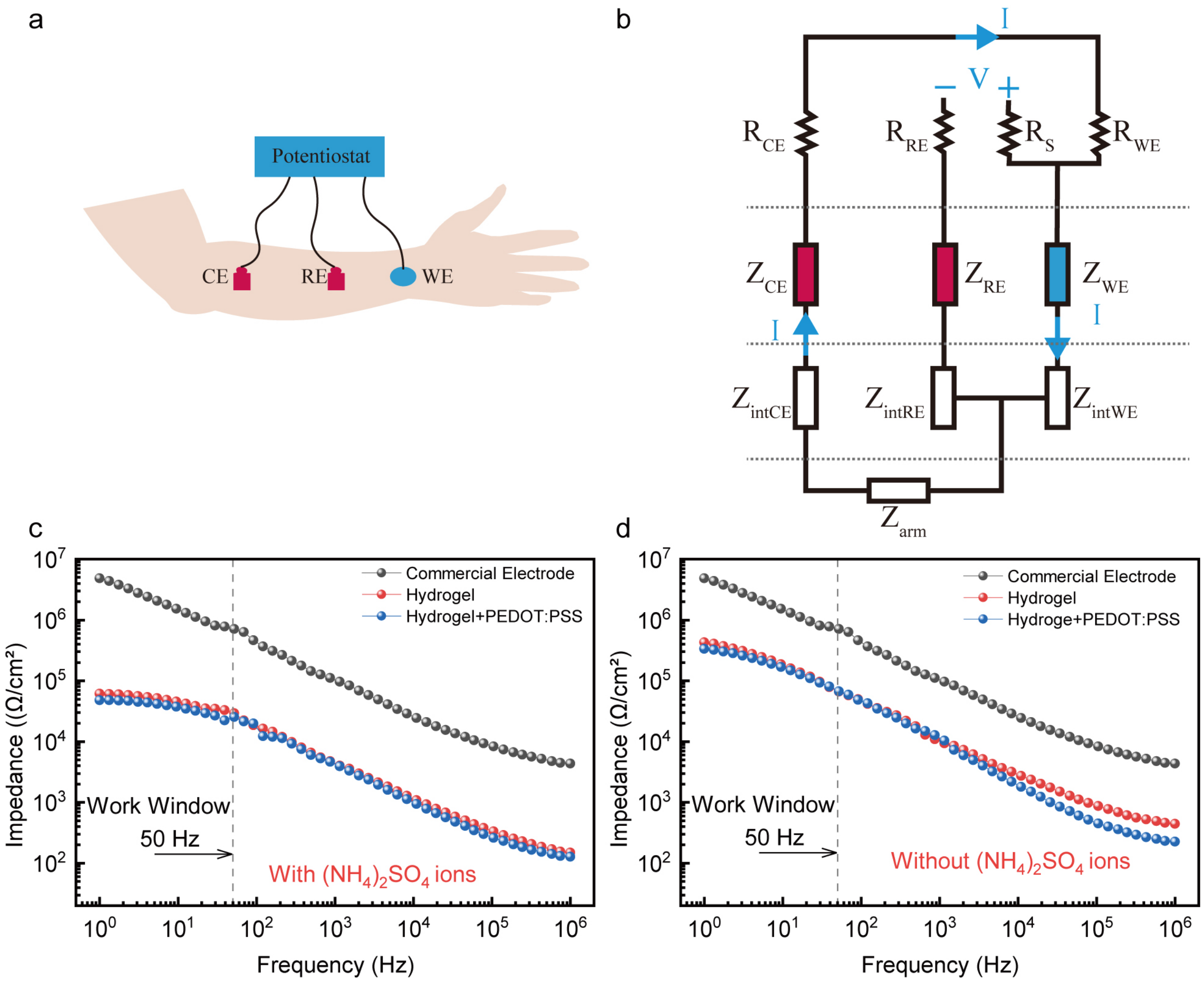

Supplementary Figure 12. PolaTex-skin interfacial impedance between PEDOT: PSS-based hydrogel and skin. (a) Schematic diagram of the testing scenario. **(b)** Schematic diagram of the equivalent circuit for testing. **(c)** Interfacial impedance measurement of hydrogel after ionic treatment. **(d)** Interfacial impedance measurement of hydrogel before ionic treatment.

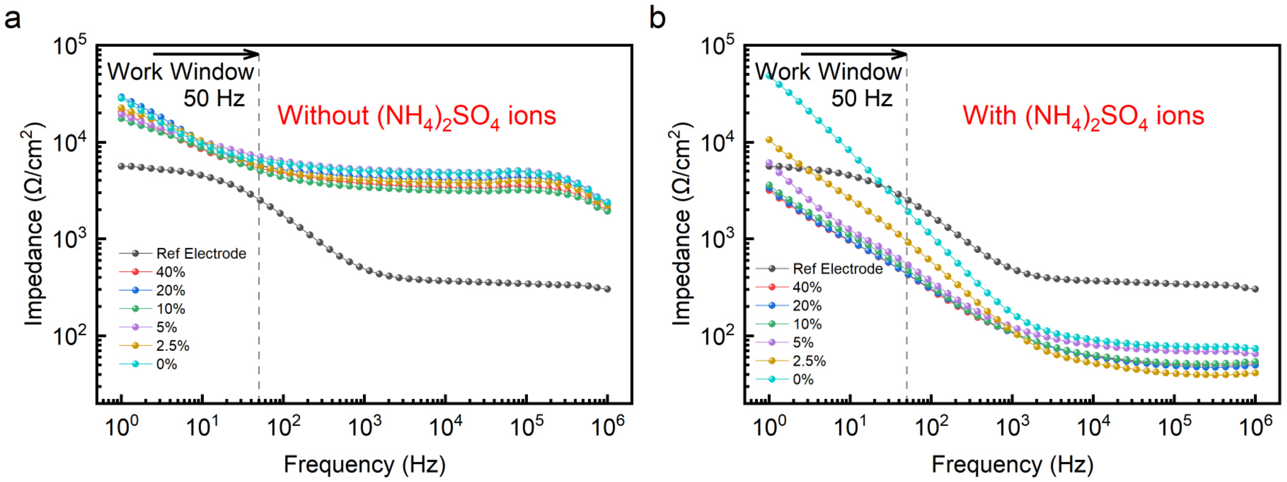

Supplementary Figure 13. Self-impedance measurement of PolaTex. (a) Impedance of body PolaTex with different PEDOT: PSS concentrations **(b)** Impedance of PolaTex with different PEDOT: PSS concentrations after ionic (20 wt% ammonium sulfate) treatment.

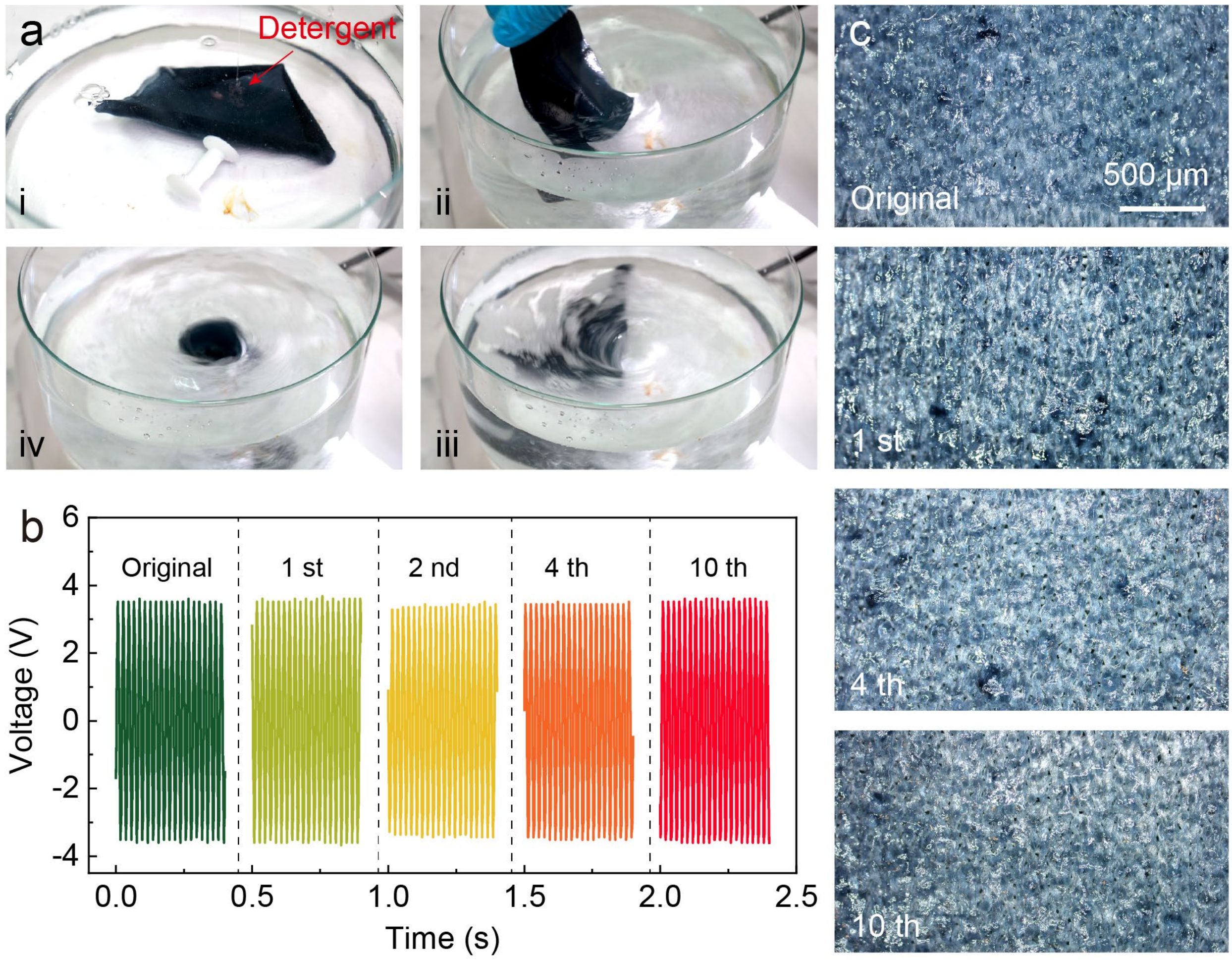

Supplementary Figure 14. Washability of Pola-tex. (a) Digital photo of the Pola-Tex washing process. **(b)** EM energy recovery performance of Pola-Tex before and after washing. **(c)** Microscopic morphology of the hydrogel layer on the surface of Pola-Tex before and after washing. The images provided here are representative of micrographs obtained from three samples.

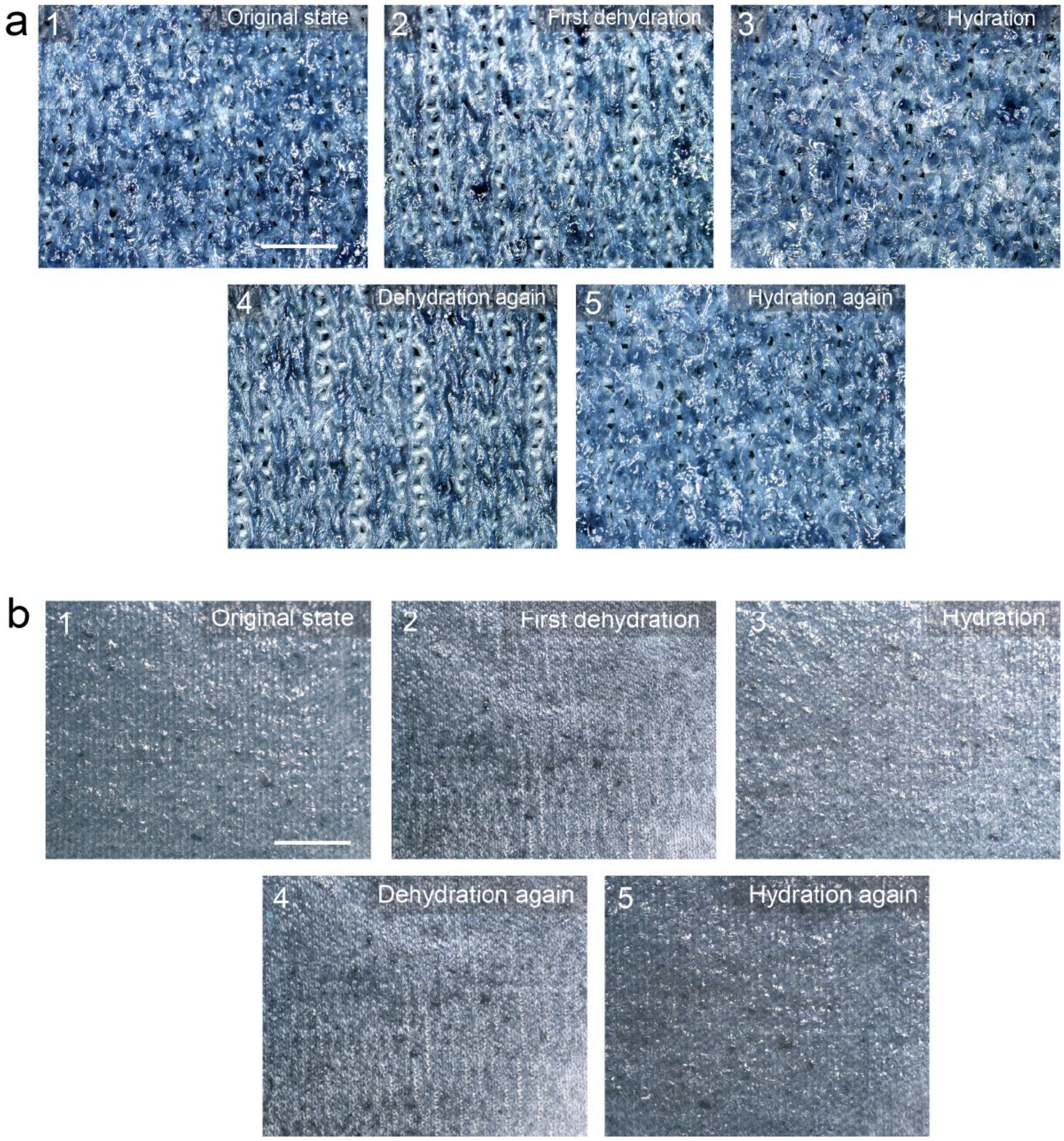

Supplementary Figure 15. (a) Optical microscopy and **(b)** digital photographs of hydrogel dehydration and its water recovery properties. Scale bar in **(a)** is 1 mm and **(b)** is 2.5 cm. The images provided here are representative of micrographs obtained from three samples.

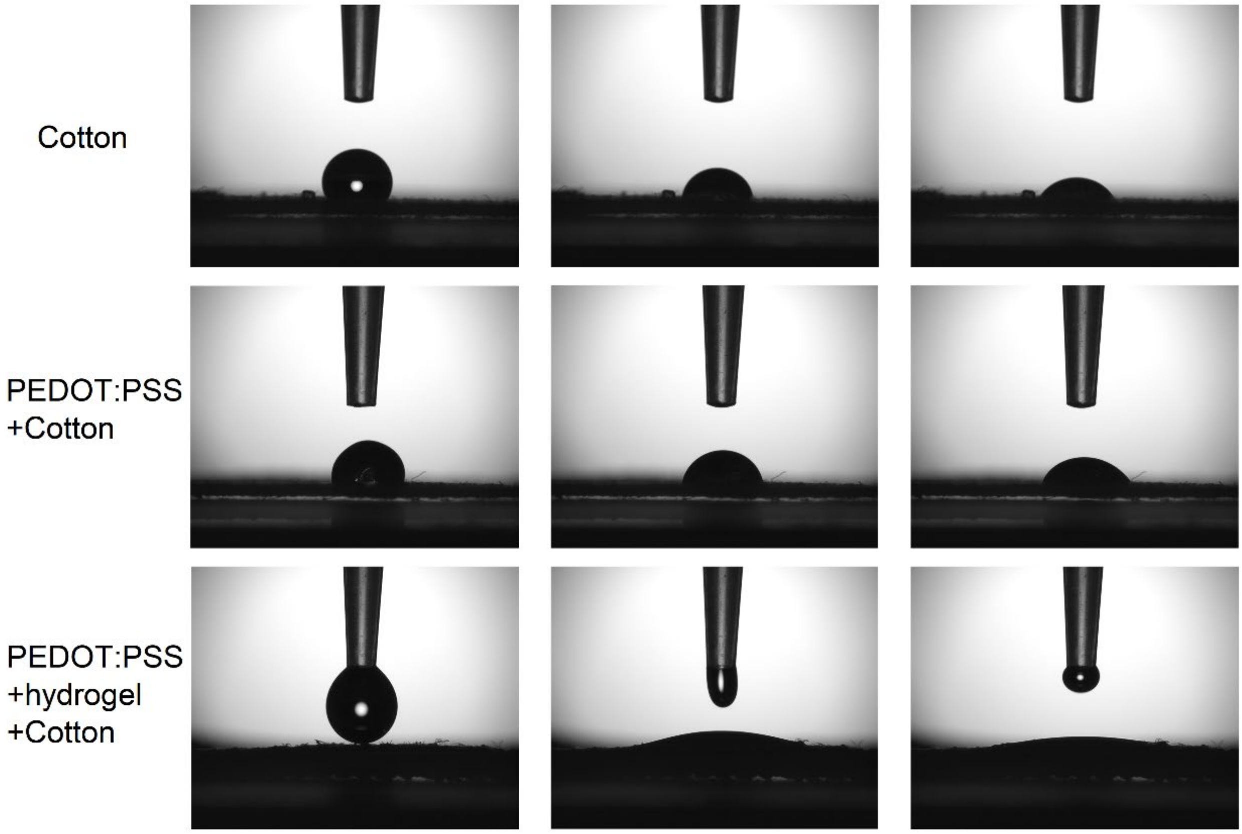

Supplementary Figure 16. The contact angle tests of PolaTex.

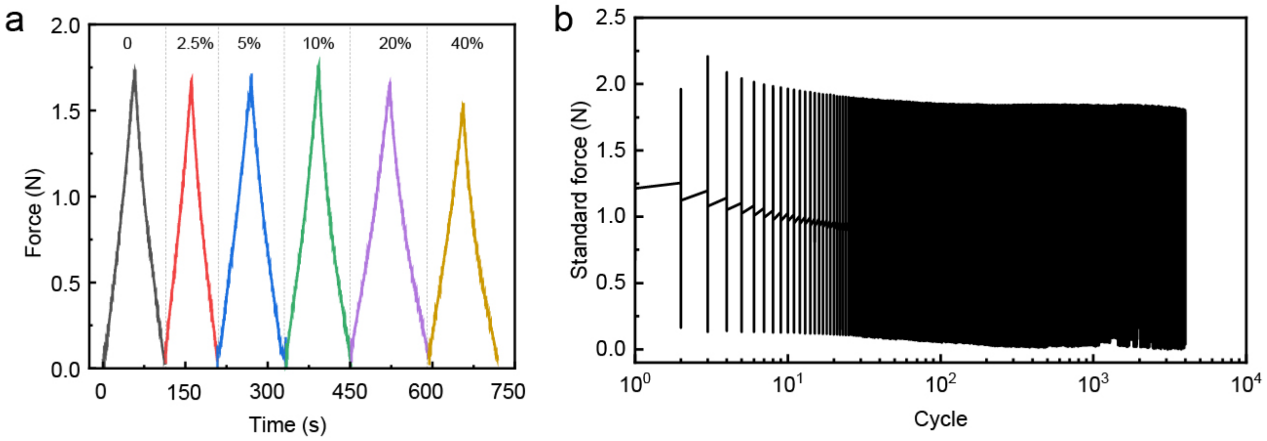

Supplementary Figure 17. Mechanical performance testing of hydrogel. (a) The stress-strain curves of hydrogels with different PEDOT: PSS doping concentrations. **(b)** The stress response of hydrogel under 4000 cyclic compression tests.

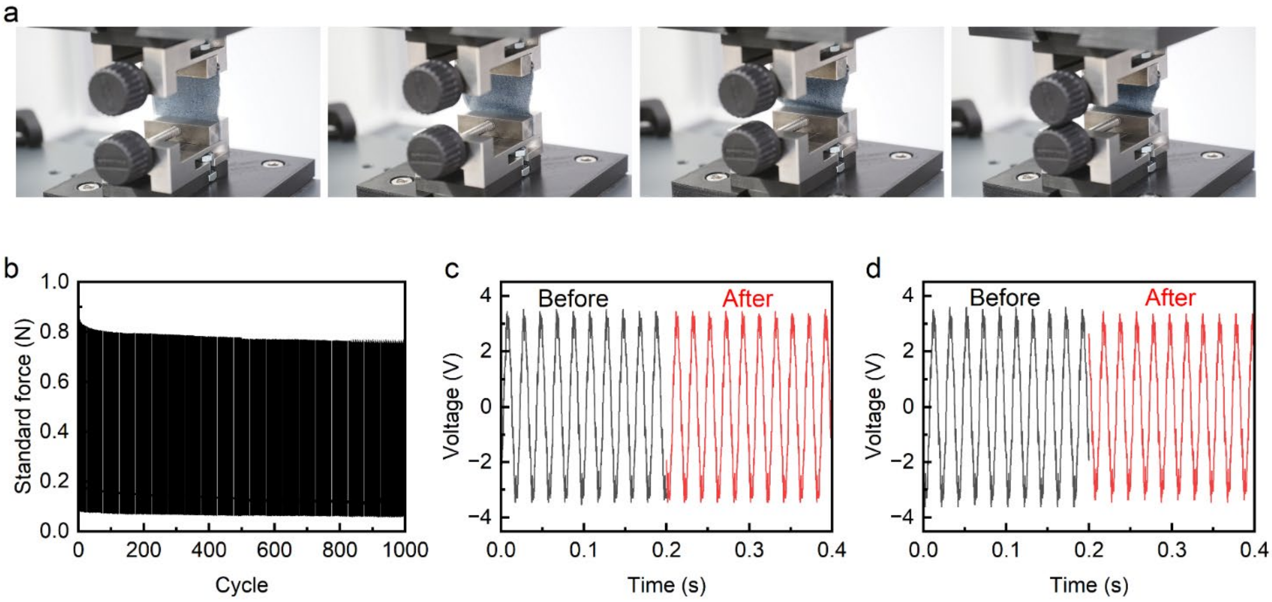

Supplementary Figure 18. Mechanical cyclic stability of the PolaTex. (a) Digital photo of the PolaTex bending cycle test process. **(b)** Mechanical data of PolaTex bending cycle under 1000 cycles. **(c)** Electrical output performance of PolaTex after 1000 bending cycles. **(d)** Electrical output performance of PolaTex after 1000 compression cycles.

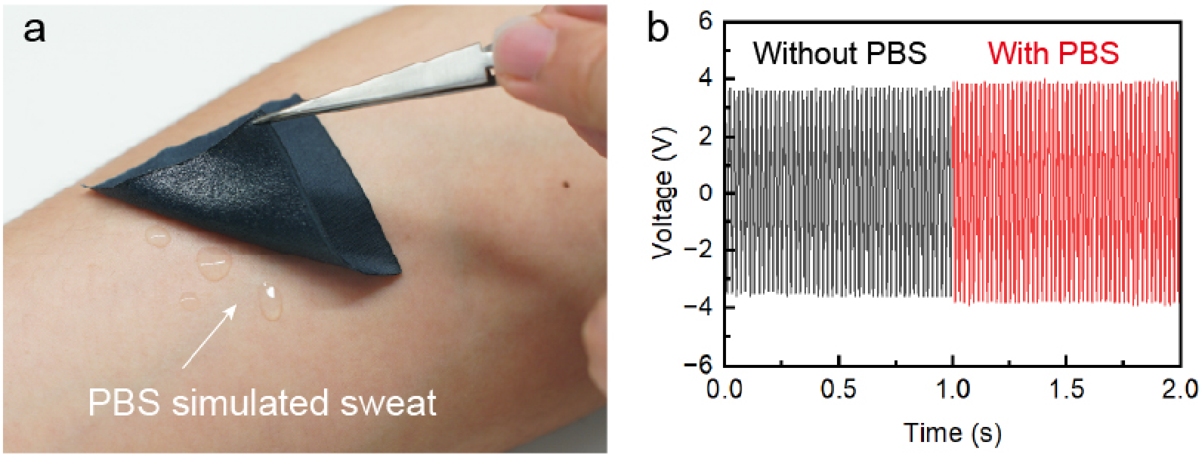

Supplementary Figure 19. (a) Digital photo of PBS simulating sweat dripping on the skin surface. **(b)** Comparison of EM energy recovery by PolaTex before and after adding PBS simulated sweat.

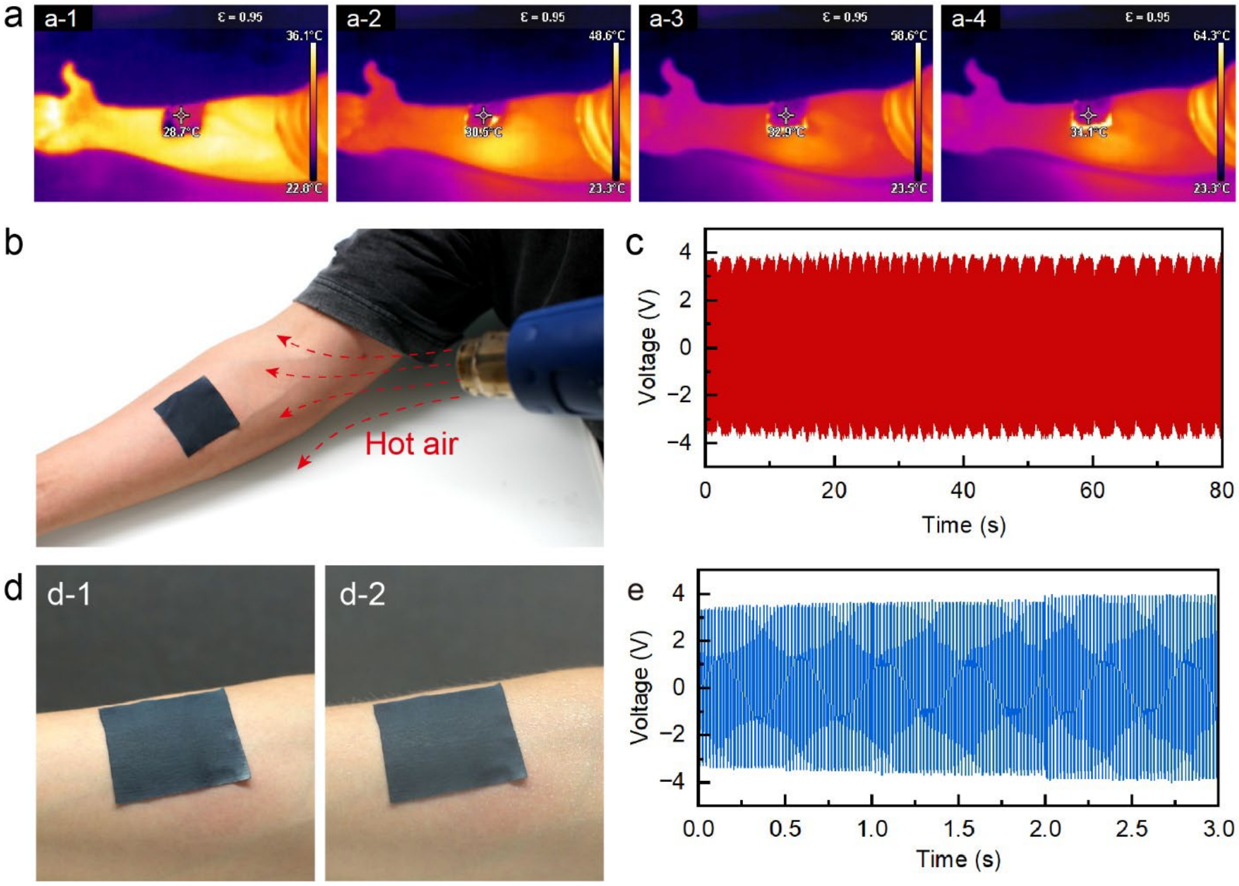

Supplementary Figure 20. Effect of **(a-c)** High temperature and **(d, e)** high humidity on electrical performance of PolaTex.

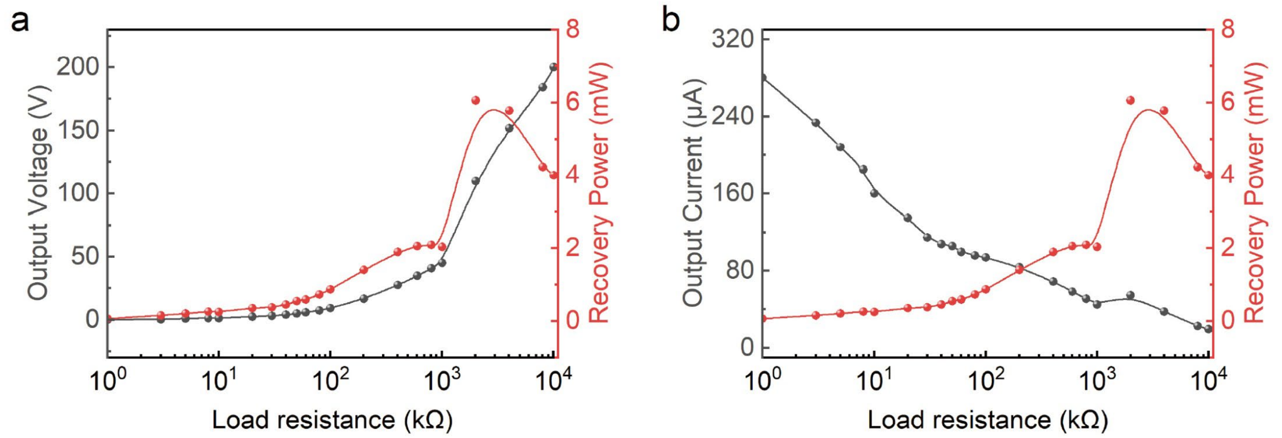

Supplementary Figure 21. The output characteristic curves under different loads. (a) The output voltage and output power curves under different loads. **(b)** The current output and output power curves under different loads. The signal was measured during daily office typing while touching the laptop.

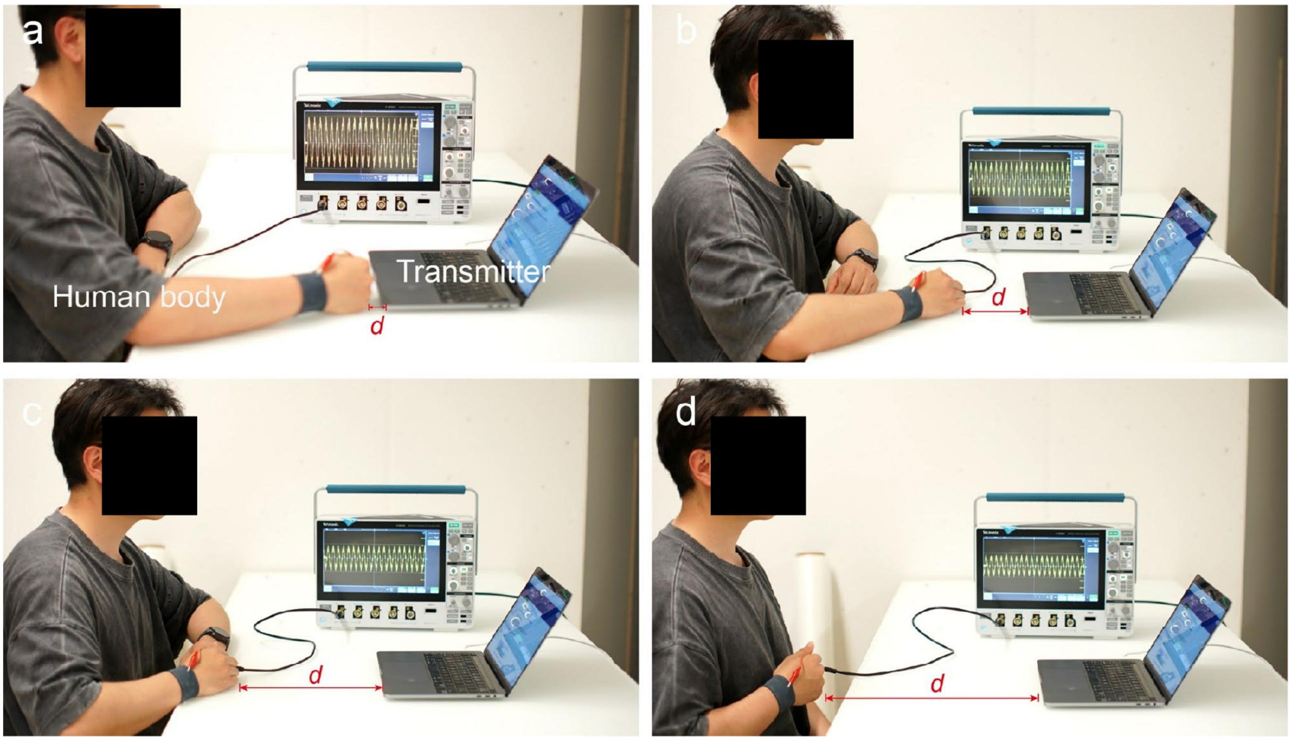

Supplementary Figure 22. Voltage and spectrum testing of EM energy recovery based on PolaTex. (a-d) Different distances between the human body and the transmitter.

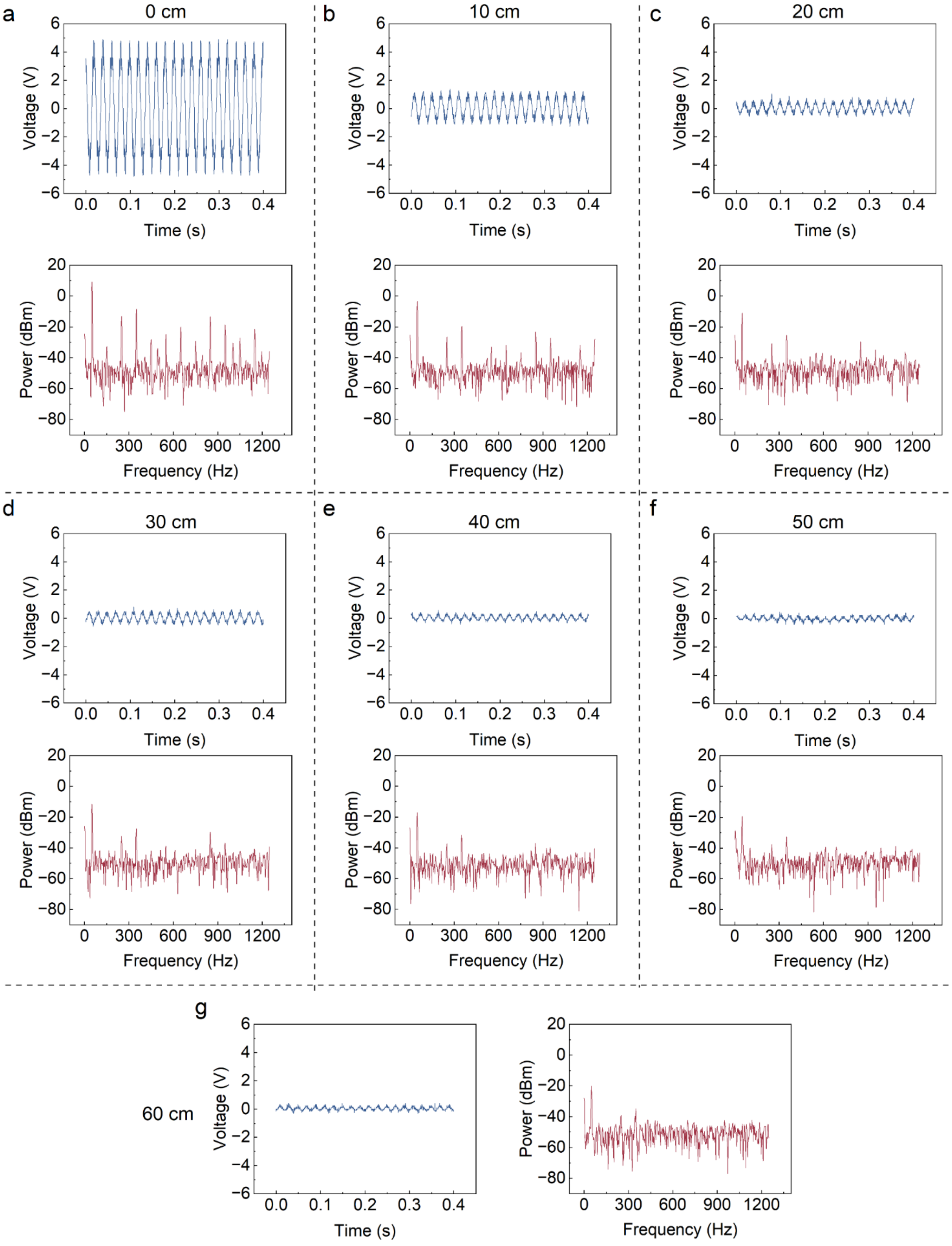

Supplementary Figure 23. Comparison of the recovered voltage at different distances in an EM field environment. Performance of the recovered electrical signal in the time and frequency domains at different distances in an electromagnetic field environment: **(a)** touch; **(b)** 10 cm; **(c)** 20 cm; **(d)** 30 cm; **(e)** 40 cm; **(f)** 50 cm; **(g)** 60 cm.

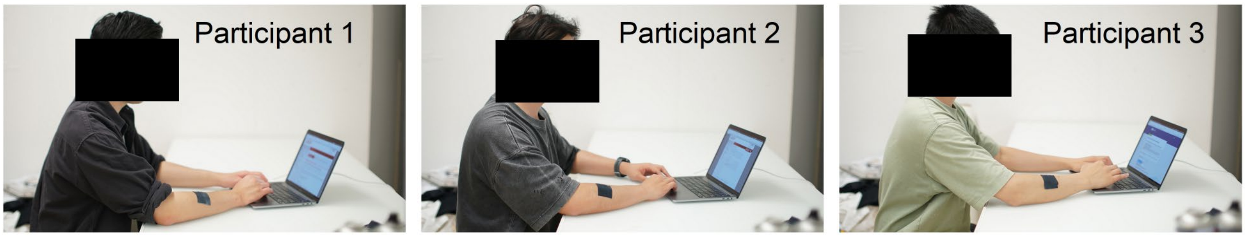

Supplementary Figure 24. Digital photos of different participants recovering EM energy through PolaTex.

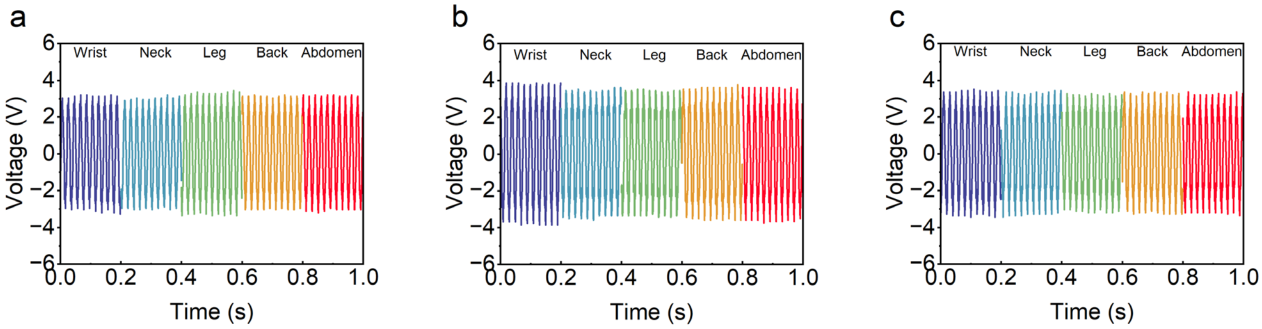

Supplementary Figure 25. Comparison of the output voltage recovered at different body locations of different participants. Voltage output measured at different body locations for participants **(a)**, **(b)** and **(c)**.

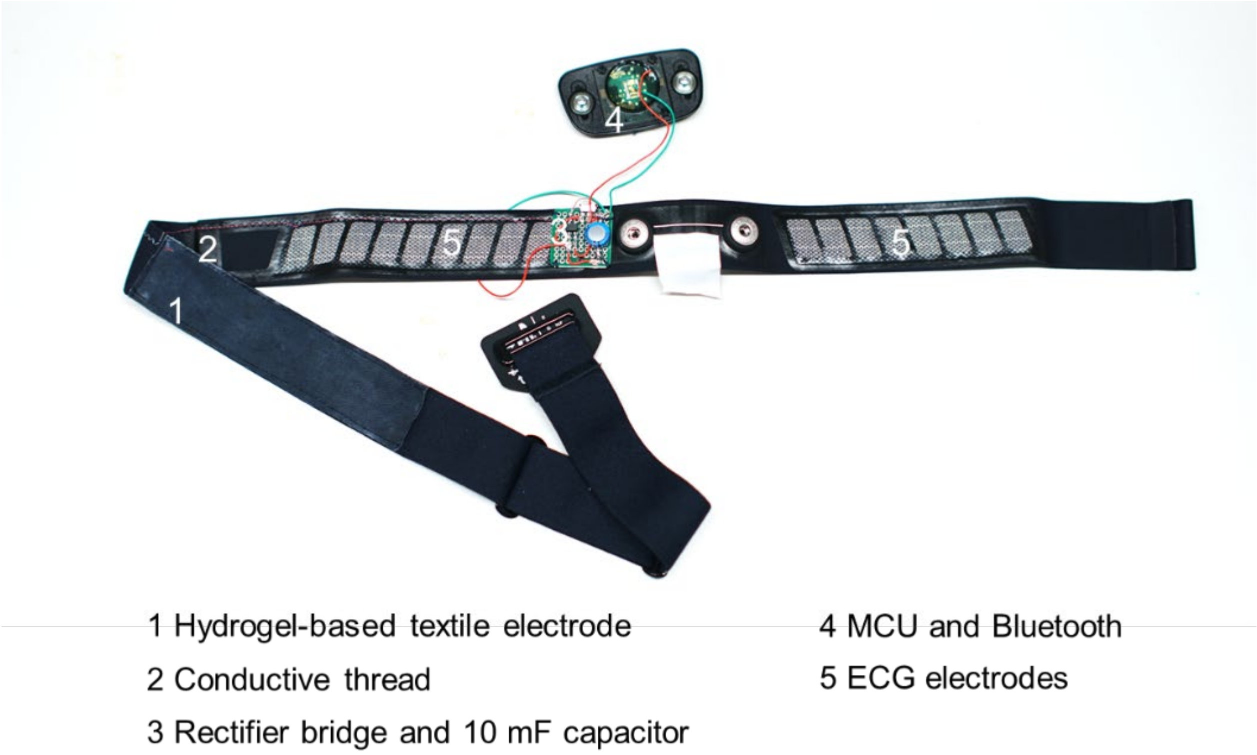

Supplementary Figure 26. PolaTex is integrated with heart rate band.

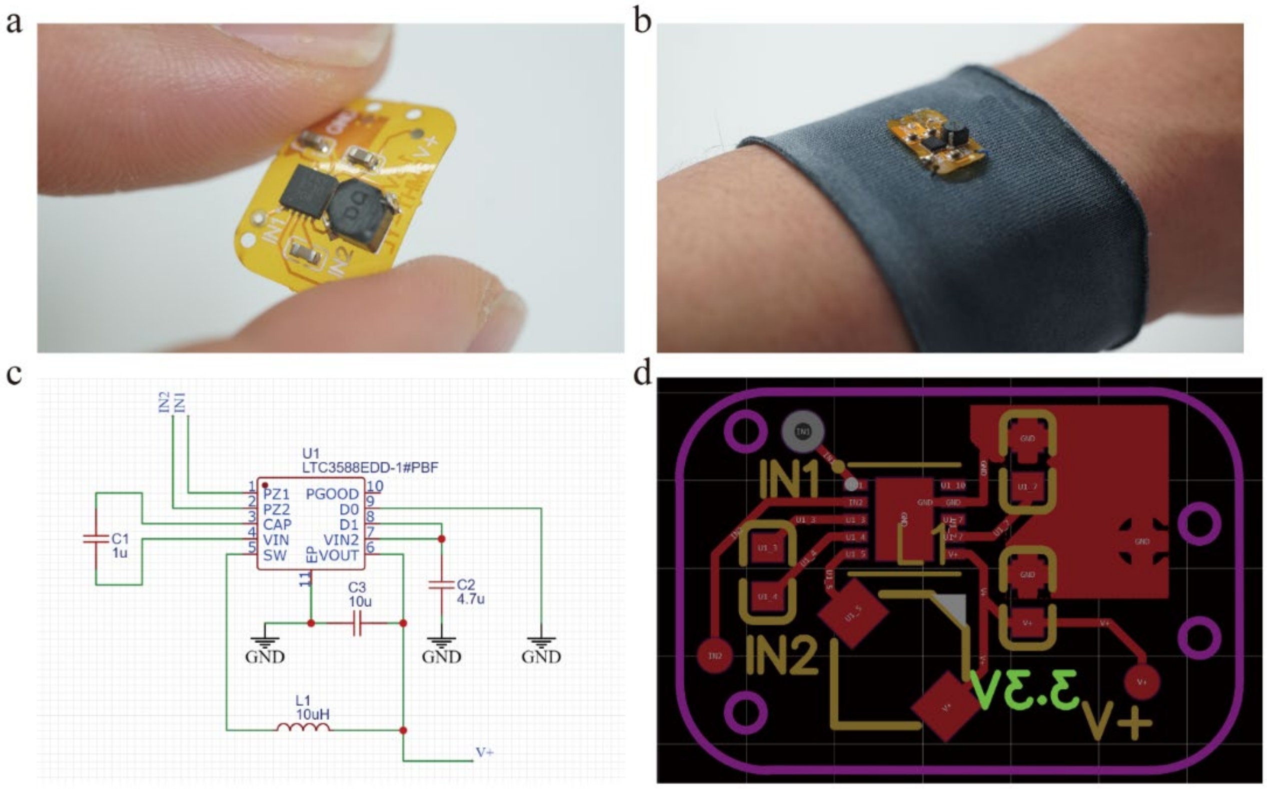

Supplementary Figure 27. The flexible energy management circuit. (a) The physical demonstration of the flexible circuit. **(b)** The integration of the flexible energy management circuit with the wristband. The schematic diagram **(c)** and PCB layout **(d)** of the flexible energy management circuit.

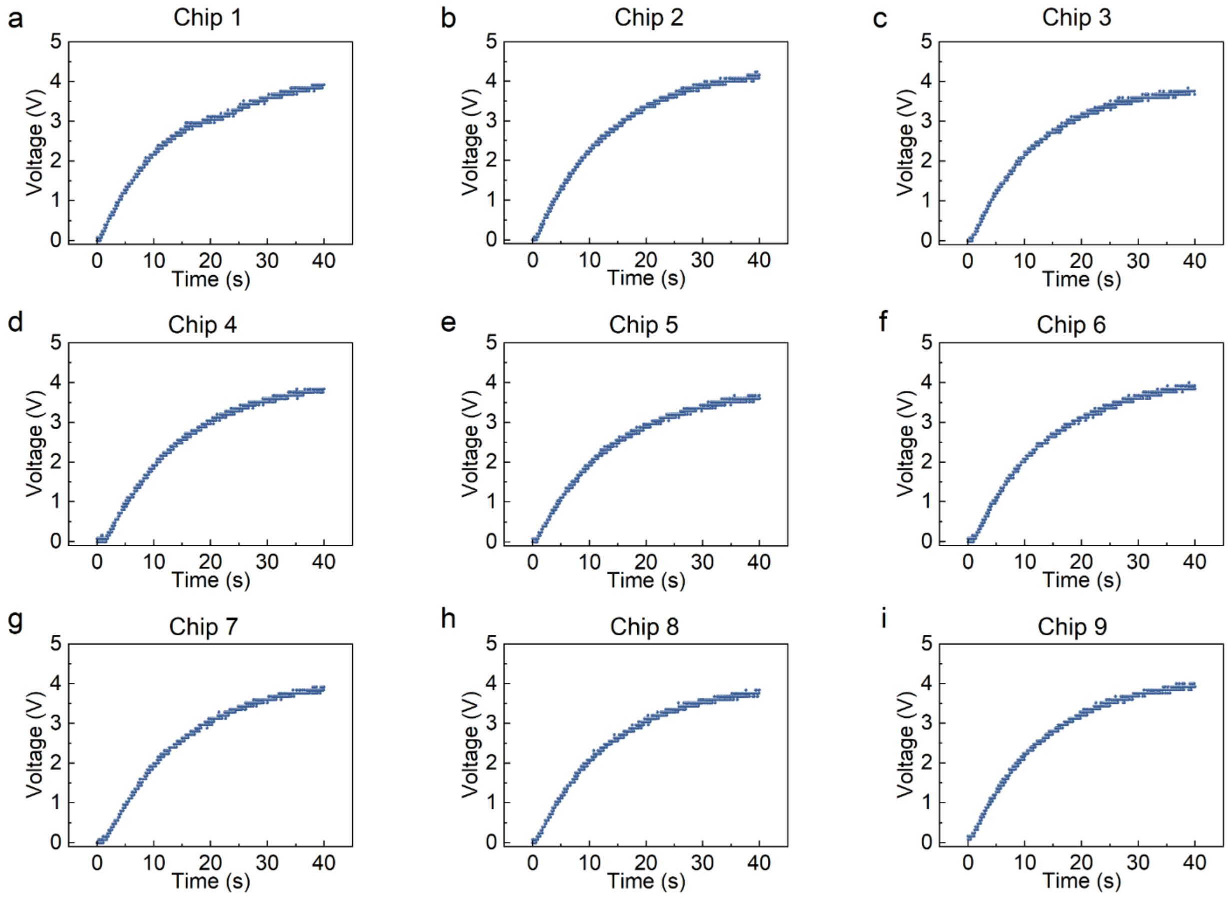

Supplementary Figure 28. Comparison of different rectifier bridges for capacitors charging in an EM environment.

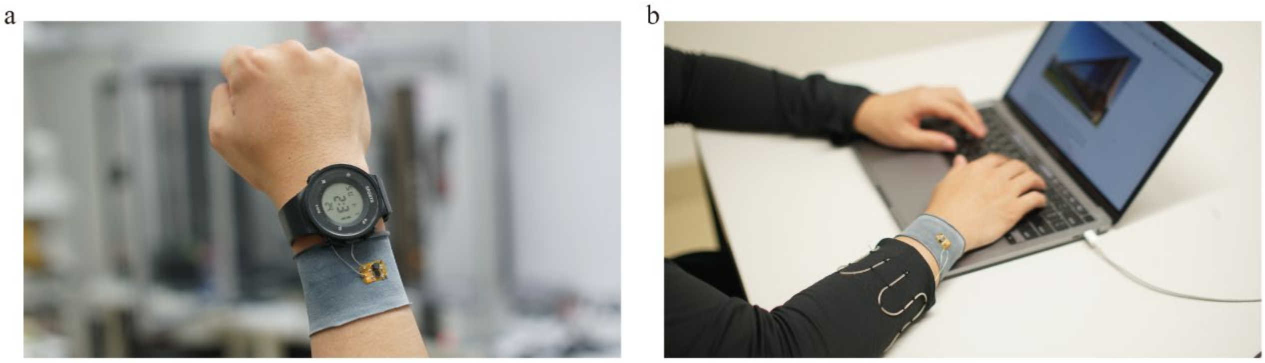

Supplementary Figure 29. The application demonstration of the PolaTex wristband. (a) The PolaTex wristband for powering a smartwatch. **(b)** The PolaTex wristband for powering luminous fibers.

Supplementary Tables

Supplementary Table 1. Comparison of existing energy supply strategies for wearable electronics.

	Principle	Frequency	Power density/ efficiency	Flexibility	Body Integrability	Ref
**EM energy harvesting (Low *f*)**	**Body polarization**	**50 Hz**	**10.01 dBm; 10.2 mW/person while daily typing; 11.02 µW at 50 cm**	**Soft textile; breathable**	**Textile at any body position**	**Our work**
**EM energy harvesting (Low *f*)**	Body-coupling	30-90 MHz; 50/60 Hz	2 µW at 160 cm	Rigid PCB; airtight	Fixed at specific skin location	[1]
	Body-coupling	0-6 Hz; 50-60 Hz	10~15 mV/mm	Rigid PCB; airtight	Fixed at specific skin location	[2]
**EM energy harvesting (High *f*)**	Schottky rectifiers	2.4 GHz	2% at −20 dBm	Soft film; airtight	Not shown	[3]
	Spin rectifiers	2.4 GHz	7.8% at −27 dBm	Rigid PCB; airtight	Non-conformal	[4]
	Schottky rectifiers	2.4 GHz	64.4% at a 10 dBm	Textile with rigid diodes; breathable	Fixed at specific textile location	[5]
**Wireless energy transmission**	Inductive coupling	13.56 MHz	~62% contacted; **~2% at 5 cm**	Textile coil; breathable	Fixed at specific textile location	[6]
**Ambient energy harvesting**	Triboelectric	3 Hz	66.13 mW m^{-2} at 10 MΩ	Soft textile; breathable	Fixed at specific textile location	[7]
		DC	0.287 mW m^{-2} at 300 kΩ	Soft textile; breathable	Fixed at specific textile location	[8]
	Photovoltaic	DC	16 %; 10 mW cm^{-2}	Soft film; airtight	Fixed at specific skin location	[9]
	Thermoelectric	DC	20.29 µW cm^{-2} K^{-2}	Soft film; airtight	Fixed at specific skin location	[10]
			0.981 µW cm^{-2} K^{-2}	Textile with rigid pillars; breathable	Fixed at specific textile location	[11]

Supplementary Table 2. Summary of research on the potential impact of low-frequency electromagnetic fields on human health.

Aspects of impact	Experimental conditions	Animal species	Main effect	Ref
Immune	50 Hz 100 µT for 20 h/day for 12 weeks	Mice	No significant effects on peripheral hematopoietic system	[12]
	50 Hz 7 mT 24 h	Male mice	Significant increase of blood parameters, such as white blood cells, lymphocytes, hemoglobin, and hematocrit levels.	[13]
Cell reproduction	50 Hz 25 µT for 18 weeks	Male mice	Increase in serum levels of male luteinizing hormone after 18 weeks; Decreased in testosterone levels after 6 and 12 weeks	[14]
	60 Hz 8.8 µT for 72 h	Mice	Low sperm counts for 72 h exposed animals, without altering male germ cell morphological characteristics.	[15]
Cancer	50 Hz 2mT	Mice	No obviously association	[16]
	60 Hz 2mT for 29 weeks	Mice	No obviously association	[17]
Cardiovascular	50 Hz 1µT	Mice	No obviously association	[18]
	60 Hz 2.4mT for 2h	Mice	The reduction of glutathione content in the heart	[19]
Nervous system	40 Hz 7mT for 15min/day for 4 weeks	Brain stroke patients	Oxidative stress can be modulated by EM field.	[20]
	50 Hz 1mT	Mice	Enhancement of hippocampal neurogenesis and proliferation of embryonic neural stem cells.	[21]

Supplementary Table 3. Summary of electromagnetic energy recovery by rectifier bridge chips composed of different diodes.

Chip NO.	Chip Name	Typical Reverse Current	Junction Capacitance	Forward Voltage
1	ABS10	5 μA	10pF@Vr=4V f=1MHz	820mV@100mA
2	KMB220S	0.05mA	28pF@Vr=4V f=1MHz	900mV@2A
3	EABS1D	1 μA	10.8pF@Vr=5V f=1MHz	950mV@1A
4	CDBHM2100	1 mA	125pF@Vr=4V f=1MHz	600mV@100mA
5	BAR6303	10 nA	0.21pF @ Vr=5V f=1MHz	950mV@100mA
6	Sstpad5 sot-23	5 pA	1.5pF@Vr=5V f=1MHz	800mV@5mA
7	CMAD 6001	500 pA	2pF@Vr=0V f=1MHz	1.1V@100mA
8	KBP310	5 μA	11.3pF@Vr=5V f=1MHz	1.1V@3A
9	DBL106G	2 μA	25pF@Vr=4V f=1MHz	1.1V@1A

Supplementary Notes

Supplementary Note 1 Comparison of existing energy supply strategies for wearable electronics. To highlight the advantages of body dielectric polarization in energy recovery, body integrability, and comfort, we compared it with existing energy supply solutions for body-area sensor network systems. These include low-frequency and high-frequency EM energy harvesting, wireless energy transmission, and ambient energy harvesting technologies (triboelectric, photovoltaic, and thermoelectric methods).

In terms of power density, our PolaTex achieves an EM energy recovery of 10.01 dBm at 10 cm, delivering 10.2 mW per person while daily typing. This significantly outperforms both low-frequency and high-frequency EM energy harvesting solutions. In near-field energy transmission, efficiency declines sharply as distance increases, requiring the transmitter and coil to be perfectly aligned for stable operation. Meanwhile, ambient energy harvesting technologies present their own limitations: triboelectric nanogenerators (TENGs) can achieve peak power densities in the milliwatt range but require an external high-impedance (GΩ level) load and are highly sensitive to humidity. Flexible thermoelectric devices, on the other hand, generally operate at the microwatt level, limiting their practical application.

In terms of wearing comfort and body integration, our PolaTex offers a key advantage—it directly recovers energy from the human body using only conductive textile electrodes, eliminating the need for additional power generation materials. Unlike traditional solutions that rely on rigid components such as diodes and inductors, our design enables energy recovery from anywhere on the body, without being constrained by the placement of a dedicated power generation unit. This not only facilitates distributed energy acquisition but also ensures superior wearing comfort.

Supplementary Note 2 COMSOL simulation setup

To investigate the interaction between low-frequency electromagnetic fields and the human body, we performed simulations using *COMSOL Multiphysics* with the **"Electric Currents"** physics interface. This module allows us to account for both ionic space charge polarization and molecular dipole polarization, which are crucial for accurately modeling the combined effects of electrical conductivity and permittivity in biological tissues and surrounding media. Both cross-sectional and 3D field plots were generated to visualize the spatial distribution of the field around and inside the body (Supplementary Fig. 2).

1. Model Selection: Electric Currents (EC module); Frequency Domain (50 Hz)
2. Geometry: A 3D human body model was imported into COMSOL to represent a standing individual. A point source transmitter was placed at a fixed distance in front of the body to simulate a low-frequency electric field emission source (100 V, 50 Hz).
3. Material properties: Air: $\varepsilon = 1$ at 50 Hz; $\sigma = 1 \times 10^{-14}$ S/m; Human body: $\varepsilon = 1 \times 10^{6}$ at 50 Hz; $\sigma = 0.1$ S/m
4. Boundary and source settings: The transmitter was set as an electric potential boundary with 100 V amplitude and 50 Hz sinusoidal excitation. All external boundaries were set to electrical insulation (zero normal current flow).
5. Mesh settings: A fine tetrahedral mesh was applied, particularly around the body–air interface and the source region, to ensure spatial resolution of electric field gradients.
6. Postprocessing: Electric potential distribution (V) and Electric field intensity (E-field, V/m) throughout the human body and surrounding air.

Supplementary Note 3 Safey analysis of the 50 Hz EM field on human health

We believe that the impact of EM energy in our daily life environment on the human body can be mainly divided into (1) Joule heat effect and (2) electromagnetic radiation effect. The main analysis is as follows:

Impact of Joule heating effect: The EM field frequency used in our study was in the low-frequency range (50 Hz), with a maximum power output of approximately 10.01 dBm (~10.2 mW). At this power level, significant Joule heating effects on the human body are unlikely to occur.

Impact of EM radiation effect: In our research, PolaTex was used to recover extremely low-frequency EM fields at 50 Hz. Throughout the experiments, no additional signal transmitter was introduced to enhance the EM signals; only the naturally occurring EM fields emitted by daily electrical appliances were collected. Typically, the operating voltage of common household devices is around 200 V, such as 230 V/50 Hz in Switzerland. According to the standards set by the International Commission on Non-Ionizing Radiation Protection (ICNIRP), the permissible occupational exposure limit for a 50 Hz EM field is 5 kV/m [Health Phys., 2010, 99: 818–836]. In other words, the EM environment surrounding the human body in our experiments was far below the 5 kV/m exposure threshold. On the other hand, several studies have specifically investigated the potential biological effects of low-frequency electromagnetic fields on human health ([1] Frontiers in Neuroscience, 2023, 17: 1247021; [2] PLOS ONE, 2013, 8(9): e72944; [3] Mutation Research/Genetic Toxicology and Environmental Mutagenesis, 2005, 583(2): 184–188). A summary of representative research findings is provided in the table below.

Description of Additional Supplementary Files

File Name: **Supplementary Movie 1**

Description: Recovery voltage of EM energy from different polarity dielectrics.

File Name: **Supplementary Movie 2**

Description: Preparation of the hydrogel-based breathable textile electrode. Section 1: Treatment of cotton with PEDOT:PSS; Section 2: Deposition of low *Z* hydrogel interface.

File Name: **Supplementary Movie 3**

Description: Body dielectric polarization-enabled EM energy recovery for Heart Rate Monitor (HRM). Section 1: Setup of the HRM; Section 2: Charging process of the HRM; Section 3: Connection of the HRM.

File Name: **Supplementary Movie 4**

Description: Body dielectric polarization-enabled EM energy recovery for powering light-emitting fibers. Section 1: Side view of the test; Section 2: Top view of the test.

File Name: **Supplementary Movie 5**

Description: Body dielectric polarization-enabled EM energy recovery for powering smart watch Section 1: Device setup; Section 2: Power smart watch.

Reference

[1] Li, Jiamin, et al. Body-coupled power transmission and energy harvesting. ***Nat. Electron.,*** 4.7 (2021): 530-538.

[2] Jung, Sungwon, et al. Body-mediated bioelectronics for zero-powered ion release and electrical stimulation. ***ACS Energy Lett.*** 7.11 (2022): 3997-4004.

[3] Zhang, Xu, et al. Two-dimensional MoS2-enabled flexible rectenna for Wi-Fi-band wireless energy harvesting. ***Nature*** 566.7744 (2019): 368-372.

[4] Sharma, Raghav, et al. Nanoscale spin rectifiers for harvesting ambient radiofrequency energy. ***Nat. Electron.*** 7.8 (2024): 653-661.

[5] Zada, Muhammad, et al. Battery-free digitally embroidered smart textile energy harvester for wearable healthcare IoTs. ***IEEE Trans. Ind. Electron.*** 71.8 (2023): 9865-9874.

[6] Lin, Rongzhou, et al. Wireless battery-free body sensor networks using near-field-enabled clothing. ***Nat. Commun.*** 11.1 (2020): 444.

[7] Xu, Fan, et al. Scalable fabrication of stretchable and washable textile triboelectric nanogenerators as constant power sources for wearable electronics. ***Nano Energy*** 88 (2021): 106247.

[8] Li, Yuanlong, et al. Metal-organic framework-based tribovoltaic textile for human body signal monitoring. ***Adv. Sci.*** 12.17 (2025): 2414086.

[9] Saifi, Sakeena, et al. An ultraflexible energy harvesting-storage system for wearable applications. ***Nat. Commun.*** 15.1 (2024): 6546.

[10] Liu, Yuan-Meng, et al. Boosting thermoelectric performance of single-walled carbon nanotubes-based films through rational triple treatments. ***Nat. Commun.***15.1 (2024): 3426.

[11] Jing, Yuanyuan, et al. Scalable manufacturing of a durable, tailorable, and recyclable multifunctional woven thermoelectric textile system. ***Energy Environ. Sci.*** 16.10 (2023): 4334-4344.

[12] Zhang, Y., et al. Effects of extremely low frequency electromagnetic fields on the hematopoietic system of the SD rats. ***High Voltage Eng.*** 42 (2016): 2519-2527.

[13] Wyszkowska, Joanna, et al. Evaluation of the influence of in vivo exposure to extremely low-frequency magnetic fields on the plasma levels of pro-inflammatory cytokines in rats. ***Int. J. Radiat. Biol.*** 94.10 (2018): 909-917.

[14] Al-Akhras, Moh'd-Ali, Homa Darmani, and Ahmed Elbetieha. Influence of 50 Hz magnetic field on sex hormones and other fertility parameters of adult male rats. ***Bioelectromagnetics*** 27.2 (2006): 127-131.

[15] Heredia-Rojas, J. Antonio, et al. In vivo Cytotoxicity induced by 60 Hz electromagnetic fields under a high-voltage substation environment. ***Sustainability*** 10.8 (2018): 2789.
[16] Galloni, Paolo, and Carmela Marino. Effects of 50 Hz magnetic field exposure on tumor experimental models. ***Bioelectromagnetics*** 21.8 (2000): 608-614.
[17] McLean, J. R., et al. A 60 Hz magnetic field does not affect the incidence of squamous cell carcinomas in SENCAR mice. ***Bioelectromagnetics*** 24.2 (2003): 75-81.
[18] Elmas, Onur, Selcuk Comlekci, and Halis Koylu. Effects of short-term exposure to powerline-frequency electromagnetic field on the electrical activity of the heart. ***Arch. Environ. Occup. Health*** 67.2 (2012): 65-71.
[19] Martínez-Sámano, Jesús, et al. "Effects of acute electromagnetic field exposure and movement restraint on antioxidant system in liver, heart, kidney and plasma of Wistar rats: a preliminary report. ***Int. J. Radiat. Biol.*** 86.12 (2010): 1088-1094.
[20] Cichoń, Natalia, et al. Extremely low frequency electromagnetic field (ELF-EMF) reduces oxidative stress and improves functional and psychological status in ischemic stroke patients. ***Bioelectromagnetics*** 38.5 (2017): 386-396.
[21] Ma, Qinlong, et al. Extremely low-frequency electromagnetic fields promote in vitro neuronal differentiation and neurite outgrowth of embryonic neural stem cells via up-regulating TRPC1. ***PloS One*** 11.3 (2016): e0150923.
