# Supplementary material for: Leveraging body dielectric polarization for ambient electromagnetic energy recovery via e-textile
Source: Nat Commun. 2025 Oct 9;16:9001. doi: 10.1038/s41467-025-64053-2 (PMC12511592; doi:10.1038/s41467-025-64053-2)
Supplement: Supplementary file 3 — Description of Additional Supplementary Information [file 41467_2025_64053_MOESM3_ESM.pdf]

Description of Additional Supplementary Files

File Name: **Supplementary Movie 1**
Description: Recovery voltage of EM energy from different polarity dielectrics.

File Name: **Supplementary Movie 2**
Description: Preparation of the hydrogel-based breathable textile electrode. Section 1: Treatment of cotton with PEDOT:PSS; Section 2: Deposition of low *Z* hydrogel interface.

File Name: **Supplementary Movie 3**
Description: Body dielectric polarization-enabled EM energy recovery for Heart Rate Monitor (HRM). Section 1: Setup of the HRM; Section 2: Charging process of the HRM; Section 3: Connection of the HRM.

File Name: **Supplementary Movie 4**
Description: Body dielectric polarization-enabled EM energy recovery for powering light-emitting fibers. Section 1: Side view of the test; Section 2: Top view of the test.

File Name: **Supplementary Movie 5**
Description: Body dielectric polarization-enabled EM energy recovery for powering smart watch Section 1: Device setup; Section 2: Power smart watch.
